# Mechanism of inhibition of acid-mediated transthyretin aggregation by designed peptides

**DOI:** 10.1016/j.jbc.2025.110594

**Published:** 2025-08-13

**Authors:** Xun Sun, Rose Pedretti, H. Jane Dyson, Lorena Saelices, Peter E. Wright

**Affiliations:** 1 Department of Integrative Structural and Computational Biology and Skaggs Institute of Chemical Biology, The Scripps Research Institute, La Jolla, California, USA; 2 Center for Alzheimer’s and Neurodegenerative Diseases, Department of Biophysics, Peter O’Donnell Jr Brain Institute, University of Texas Southwestern Medical Center, Dallas, Texas, USA

**Keywords:** transthyretin aggregation, amyloid, aggregation inhibitor, aggregation mechanism, aggregation kinetics, real-time ^19^F-NMR

## Abstract

Aggregation of transthyretin (TTR) causes TTR cardiomyopathy and polyneuropathy through amyloidosis. To initialize TTR aggregation, the native TTR tetramer first dissociates to a monomeric intermediate, which misfolds and self-assembles to oligomers, eventually forming insoluble aggregates and fibrils. Peptide inhibitors have been designed to cap two β-strands that are buried in the well-folded tetramer but are solvent-exposed in the monomeric aggregation intermediate. However, how these peptides affect the reaction kinetics of individual steps in the multi-step TTR aggregation pathway remains unknown. Here, we integrated ^19^F-NMR and kinetic modeling to determine aggregation reaction rates of individual steps with the peptide inhibitors and extract the free energy landscape along the TTR aggregation pathway. We found direct kinetic evidence that the peptide inhibitors bind to monomeric intermediates and misfolded tetramers at acidic pH, but do not bind to structured TTR monomers or tetramers at neutral pH. In addition, the peptides do not bind to amorphous aggregates formed at acidic pH and physiological temperature *in vitro*, in contrast to the previously reported findings that these peptides recognize *ex vivo* TTR fibrils derived from patients with TTR amyloidosis. Interestingly, the peptides bind to soluble oligomers formed at acidic pH and low temperature *in vitro*, suggesting that these oligomers may share structural similarity with misfolded monomeric intermediates and patient-derived TTR fibrils. Our methods provide quantitative and mechanistic details for peptide-inhibited TTR aggregation.

Transthyretin (TTR) is an abundant serum protein that transports thyroxine and retinol binding protein in blood and cerebrospinal fluid. Structurally, TTR is a tetramer, formed as a dimer of dimers (Fig. 1*A*). Each TTR protomer has a β-sandwich fold, formed by two β-sheets, each consisting of four β-strands. Under destabilizing conditions, for example, at acidic pH (1) or with pathogenic mutations (2), TTR becomes aggregation-prone and forms pathogenic aggregates, a hallmark for amyloidogenic proteins whose aggregation causes various types of amyloidosis diseases affecting a wide range of organs and tissues (3). Deposits of TTR aggregates in the heart and central nervous system can lead to devastating cardiomyopathies and polyneuropathies, respectively. In the case of wild-type (WT) TTR amyloidosis, aggregation of WT TTR leads to WT TTR amyloidosis, a restrictive cardiomyopathy affecting up to 25% of the population over the age of 80 (4).

**Figure 1:**
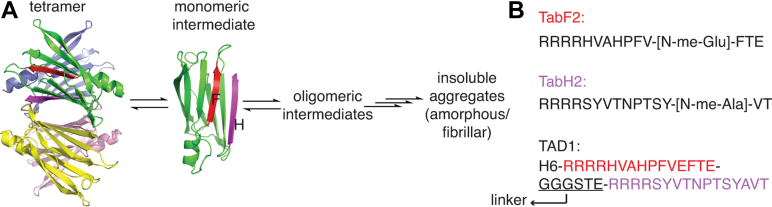
**Schematic digram of the aggregation of TTR and peptide sequences.***A*, TTR aggregation is initiated by dissociation of the tetramer to monomer, which misfolds and self-assembles to form oligomers and higher orders of aggregates along the multi-step TTR aggregation pathway. The F and H β-strands are colored in red and magenta, respectively. *B*, sequences of designed peptide inhibitors TabF2, TabH2 (5, 6) with N-methylation sites denoted, and fluorophore-free TAD1 (ffTAD1) (7) without N-methylation.

TTR aggregation is a multi-step process. To initialize the aggregation, the native TTR tetramer first dissociates to a structured monomer, which misfolds and self-assembles to soluble oligomers and eventually forms insoluble aggregates (1, 8). Morphologies of insoluble TTR aggregates are condition-dependent; factors including pH, mutations, and disease phenotypes can influence the structural fold of aggregates formed (5, 8, 9, 10).

Tafamidis, the first FDA-approved small-molecule drug for treating cardiac TTR amyloidosis, binds to the central cavity of the TTR tetramer (Fig. 1*A*). The resulting stabilization of the tetramer suppresses dissociation to monomers and reduces the TTR population entering the aggregation pathway (11). However, the efficacy of tafamidis is reduced for patients with TTR amyloidosis diagnosed at a later stage, when severe organ damage caused by TTR aggregates cannot be reversed (12). Additionally, tafamidis does not effectively inhibit seeded TTR aggregation, where extracted amyloidogenic fibrils from patients with TTR amyloidosis are used as seeds for aggregation assays *in vitro* (5). To this end, peptide inhibitors have been developed to inhibit seeded TTR aggregation. The first-generation peptide inhibitors are based on TTR sequences from the F-strand (^91^AEVVFT^96^) and H-strand (^119^TAVVTN^124^), where four arginine residues are added preceding the respective F- or H-strand sequences (13). The F and H β-strands are solvent-exposed in TTR monomers but buried in folded TTR tetramers (Fig. 1*A*). The second-generation peptides are optimized against the same F and H β-strand sequences with selected N-methylation sites and show improved efficacy (5, 6) (Fig. 1*B*). The latest version of the peptide inhibitor, ffTAD1, is a fluorophore-free derivative of TAD1, or transthyretin aggregation detector 1 (7), and it has an N-terminal His tag and concatenated TabF2 and TabH2 sequences that are separated by a flexible linker (Fig. 1*B*). TabH2/TabF2 and ffTAD1 are effective capping peptide inhibitors for seeded TTR aggregation *in vitro* (5, 6, 7, 13, 14). However, how these peptides affect reaction kinetics for individual steps in the multi-step TTR aggregation pathway remains unknown.

In this work, we use ^19^F-NMR and kinetic modeling to reveal kinetic details on how peptides reduce rates for individual steps in TTR aggregation at acidic pH. We show that the binding of peptides to the monomeric aggregation intermediate causes greater free energy reduction than binding to the misfolded tetramer. We also find direct kinetic evidence that peptides bind to soluble TTR oligomers but not to amorphous aggregates. The knowledge on how peptides target specific aggregation species and individual reaction steps could guide future development of next-generation aggregation inhibitors for TTR and other aggregation-prone proteins.

## Results

### No detectable binding of designed peptides to folded TTR tetramer and monomer at neutral pH

**Figure 2:**
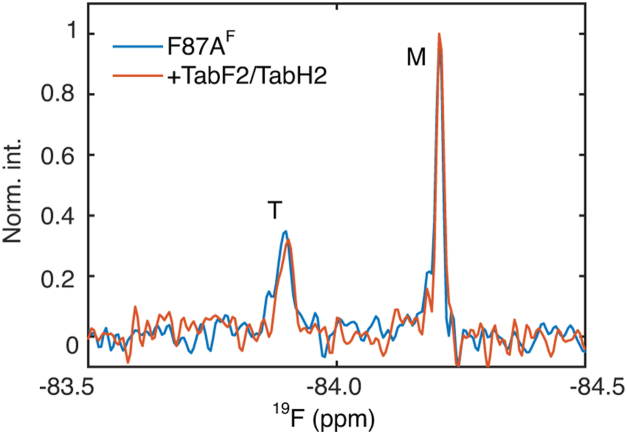
**^19^F-NMR spectra ofF87A^F^.** The protein was at 5 μM (protomer concentration) with and without TabF2 and TabH2, each at 5 μM, at 310 K and pH 7.0. Tetramer (T) and monomer (M) species are labeled.

To measure the binding of peptide inhibitors to TTR near physiological concentrations (7–21 protomer concentration or 2–5 μM tetramer in blood) (15), we chose F87A, a TTR mutant that forms a mixture of tetramer and monomer at these concentrations and neutral pH (16). By contrast, WT TTR remains predominantly tetrameric in these concentration ranges: the concentration of free TTR monomer is 35 nM at a total tetramer concentration of 2 μM, given the tetramer *K*_d_ = 9 × 10^−25^ M^3^ (17). Binding aggregation inhibitors to TTR tetramers and monomers would stabilize the respective species and alter their populations based on relative changes in free energy. Thus, the relative population of tetramer to monomer in solutions of F87A is a convenient readout of preferential stabilization of tetramer or monomer upon binding of peptides. With a highly sensitive trifluoroacetyl probe coupled to S85C, the relative population of tetramer and monomer species of F87A^F^ (notation for trifluoroacetyl-labeled C10S-S85C-F87A) can be quantified using distinct ^19^F chemical shifts by ^19^F-NMR spectroscopy (18). At a total protomer concentration of 5 μM F87A^F^, the addition of a mixture of TabF2 and TabH2 peptides at equal concentrations of 5 μM shows no noticeable population changes for F87A^F^ (Fig. 2), hinting at no detectable binding of peptides to either TTR tetramer or monomer at near physiological concentration of TTR in blood. Alternatively, the lack of relative population changes could be due to similar but weaker than 5-μM binding affinities of peptides to both tetramer and monomer species, which is unlikely due to the lack of changes in chemical shifts or increases in peak widths upon binding (Fig. 2). These data are consistent with previous work, using dot blotting with fluorescently labeled peptides in low μM ranges, which showed that the peptides do not bind strongly to structured tetramer or monomer TTR at physiological pH (14).

To probe the possible binding between TTR and peptides with residue-level resolution, we performed ^15^N-HSQC spectroscopy, which is exquisitely sensitive to weak interactions. We titrated TabF2 and TabH2 peptides at equal concentrations into ^15^N-labeled WT TTR at 56 μM tetrameric concentration with a two- or 4-fold [peptides: tetrameric WT] molar ratio (Fig. S1*A*) and observed only very small chemical shift perturbations (CSPs) for backbone amides (Fig. S2*A*). Cross peaks were shifted in fast exchange in both the ^1^H and ^15^N dimensions, indicating weak binding between the peptides and WT TTR tetramers at neutral pH. We also performed HSQC titrations for a monomeric TTR mutant F87E (19) at 106 μM (Fig. S1*B*). We observed only very small CSPs upon the addition of both peptides up to a 4-fold [peptides: monomeric F87E] molar ratio (Fig. S2*B*). The patterns of CSPs suggest the interactions between the peptides and F87E at neutral pH are weak and non-specific, as residues in the F (91–97) or H (115–122) β-strands do not show noticeable CSPs. These experiments show that the binding affinity between peptides and either tetramer or monomer at neutral pH is at least weaker than 100 μM.

### Kinetics of peptide-inhibited aggregation of TTR at acidic pH

**Figure 3:**
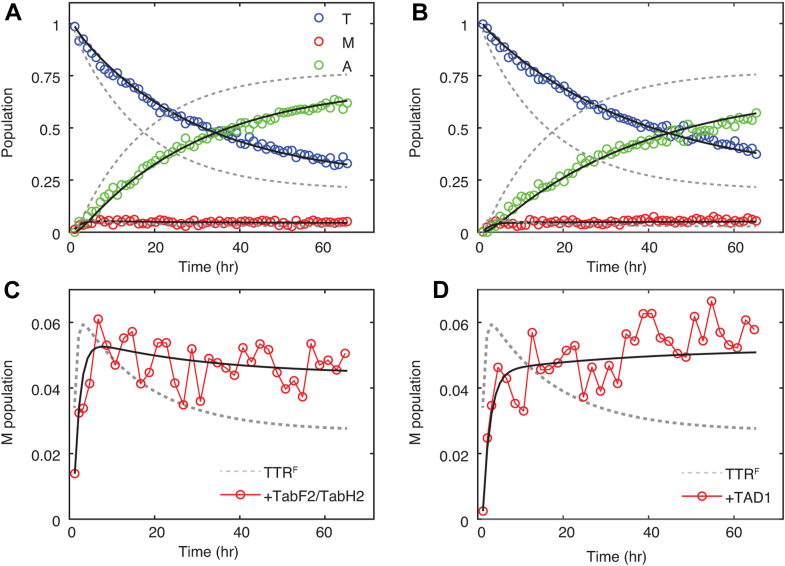
^**19**^**F aggregation kineticsat 310K.** Tetrameric TTR^F^ (2.5 μM) was incubated with 1 μM each of TabF2 and TabH2 (*A*) and 1 μM ffTAD1 (*B*) at 310 K and pH 4.4. For clarity, only the measured data points for tetramer, monomer, and aggregate signals with peptides (not the TTR^F^ data points) are shown with open circles. *Black lines* are fitted tetramer ↔ monomer ↔ aggregate models for data sets with peptide inhibitors, and *dashed gray lines* are the three-state fits for the TTR^F^-only control data for comparison. Expanded view showing the monomer population kinetics for TabF2/TabH2 (*C*) and ffTAD1 (*D*).

At acidic pH, tetramer dissociation is favored, the misfolding of the monomeric intermediate is facilitated, and the downhill aggregation is accelerated (1). We used a previously reported ^19^F-labeled TTR construct, C10S-trifluoroacetyl-S85C (denoted as TTR^F^), to quantify TTR aggregation kinetics using real-time ^19^F-NMR. At pH 4.4 without agitation, TTR^F^ aggregates at comparable rates to WT and allows direct measurement of the population of the monomeric aggregation intermediate as low as 300 nM (18). To quantify how the presence of peptides alters TTR aggregation kinetics, we added 1 μM of each TabH2 and TabF2 as well as 1 μM ffTAD1 to 2.5 μM TTR^F^ (tetramer concentration) at pH 4.4 and 310 K and monitored time-dependent population kinetics for tetramer, monomer, and NMR-invisible aggregates (Fig. 3, *A* and *B*). We mixed TabF2 and TabH2 at equal concentration since they are known to act synergistically from previous work using thioflavin T assays (6), and also seen in this work by ^19^F-NMR (Fig. S3). There are no changes in ^19^F peak positions or peak widths in the presence of TAD1 (Fig. S4), but there are time-dependent population changes: the decay of the tetramer population is slowed in the presence of the TabH2/TabF2 mixture, with a single exponential rate of 0.031 ± 0.001 h^−1^, compared to the rate of the TTR^F^-only sample of 0.062 ± 0.001 h^−1^. By comparison, ffTAD1 further reduces the tetramer decay rate to 0.025 ± 0.001 h^−1^ (Fig. 3*B*).

The kinetics of the weakly populated monomeric aggregation intermediate provide additional and critical insights into the multi-step aggregation process. For the TTR^F^ control, the population of the monomer undergoes a rapid initial increase within 3 h after the pH jump to reach a maximal concentration of ∼6% (∼600 nM for a total of 10 μM protomer concentration), then subsequently decays to a low level of ∼3% (∼300 nM). Interestingly, the decay of this intermediate species is suppressed with TabF2/TabH2, and to a greater extent with ffTAD1 (Fig. 3, *C* and *D*), indicating kinetic changes related to formation and consumption of this key aggregation intermediate.

To gain kinetic insights into the aggregation pathway, we fit the time-dependent aggregation data of TTR^F^ using a previously reported three-state kinetic model, tetramer ↔ monomer ↔ aggregate (18), where tetramer and monomer are NMR-visible, and aggregates are NMR-invisible. The use of three states is justified since no detectable soluble oligomers are observed by ^19^F-NMR at 310 K and pH 4.4, and this is the simplest three-state model (18). Using this model, fits of the time-dependent population changes determined from peak areas yield four first-order apparent rates. The fits are shown as solid black lines in Figure 3, and the extracted rates are listed in Table 1. Compared to the TTR^F^-only control (dashed gray lines), the rates of the first equilibrium step, namely the tetramer dissociation (*k*_1_) and tetramer formation (*k*_−1_) rates, are progressively decreased by the addition of TabH2/TabF2 and ffTAD1. For the second equilibrium, the aggregation rate (*k*_2_) is reduced by a similar 40 to 42% for TabH2/TabF2 and ffTAD1 compared to the TTR^F^-only control. Of note, the disaggregation rate (*k*_−2_) is unaffected by the peptide inhibitors.

**Table 1:** Aggregation kinetics for TTR^F^ (2.5 μM tetramer) with and without peptide inhibitors at pH 4.4 and 310 K

Condition	*k*_1_ (h^−1^)^a^	*k*_-1_ (h^−1^)^a^	*k*_2_ (h^−1^)^a^	*k*_-2_ (h^−1^)^a^	*γ*_2_ (h^−1^)^b^
2.5 μM TTR^F^^c^	0.10 ± 0.01	0.75 ± 0.12	0.73 ± 0.03	0.025 ± 0.002	0.061 ± 0.001
+ 1 μM TabF2/TabH2	0.039 ± 0.003	0.22 ± 0.03	0.44 ± 0.11	0.027 ± 0.010	0.035 ± 0.001
+ 1 μM ffTAD1	0.026 ± 0.001	0.13 ± 0.01	0.42 ± 0.04	0.031 ± 0.005	0.028 ± 0.001

a Data were fitted to the three-state kinetic model $T\begin{array}{c} k_{1}\\ ⇌\\ k_{-1} \end{array}M\begin{array}{c} k_{2}\\ ⇌\\ k_{-2} \end{array}A$, where T, M, and A stand for tetramer, monomeric intermediate, and NMR-invisible aggregate ensemble, respectively. The four fitted parameters are first-order apparent rates for population conversions. The uncertainty in rates was calculated as one standard deviation from 50 bootstrap datasets.

b The relative weight of the slow relaxation rate γ_2_ is defined as $\frac{\left(\sum_{i}k_{i}-\sqrt{{\left(\sum_{i}k_{i}\right)}^{2}-4\left(k_{1}k_{2}+k_{1}k_{-2}+k_{-1}k_{-2}\right)}\right)}{2}$ (*i* = 1, −1, 2, and −2). This rate is comparable to the single-exponential fits for the A signal increase; thus approximates the aggregation rate.

c Fitted parameters for tetrameric TTR^F^ are taken from Ref (18) for comparison.

### A kinetic model of reversible inhibited aggregation

We performed numerical simulations to elucidate how reversible binding of the peptide inhibitor to the various TTR species influences the rates of the individual steps in the three-state model. Binding of the inhibitor to the tetramer reduces only *k*_1_ (the only outgoing rate to consume tetramer) while having no effect on the other three rates (Fig. S5). Likewise, inhibitor binding to aggregates only reduces *k*_−2_, the only outgoing rate to consume aggregates. For the binding of inhibitor to monomer, the rates of the two processes that consume monomers (*k*_−1_ and *k*_2_) are reduced, but not *k*_1_ or *k*_−2_.

### Peptide inhibitors bind to misfolded tetramer and monomer, not NMR-invisible aggregates, at acidic pH and physiological temperature

Based on simulations of the three-state kinetic models with inhibitor binding steps (Fig. S5), the reduced *k*_1_, *k*_−1_, and *k*_2_ observed in the presence of the peptides show that inhibition results from binding to both tetrameric and monomeric states of TTR at pH 4.4 and 310 K. Importantly, the unchanged *k*_−2_ shows that the peptides do not bind TTR aggregates that are formed under these conditions.

When peptides bind to both tetramer and monomer, the pseudo steady-state equilibrium concentrations of each species depend on detailed balances among all four rates (Table 1). Given the fitted kinetics, the pseudo steady-state populations of both tetramer and monomer are increased in the presence of the peptide inhibitors, compared to the TTR^F^-only control (Table S1). As a result of mass conservation, the steady-state population of the NMR-invisible aggregates is slightly reduced by ∼6% and ∼8% for TabF2/TabH2 and ffTAD1, respectively, compared to the TTR^F^-only control (Table S1).

**Figure 4:**
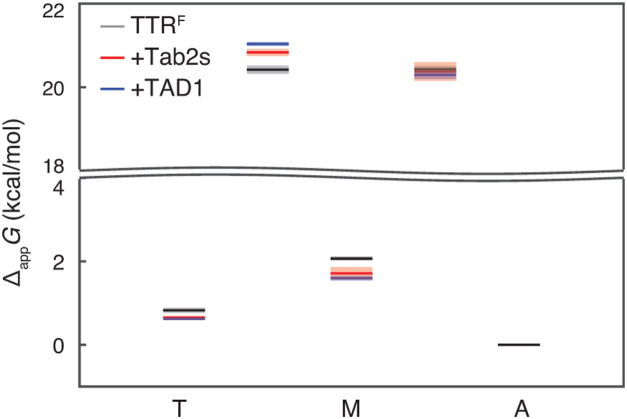
**Free energy diagram showing the effects of peptide addition.** Apparent free energy changes of tetramer and monomer at 310 K and pH 4.4 along the aggregation pathway are referenced to the aggregate state, which does not bind to peptides under these conditions. The activation energy was calculated using transition state theory. The y-axis is truncated to show energetic differences for the ground and transition states. The uncertainties in energy are shown in translucent colors.

The knowledge of inter-conversion kinetics allows us to construct an apparent free energy landscape for the aggregation pathway of TTR^F^ in the presence of peptide inhibitors at pH 4.4 and 310 K (Fig. 4). The free energy diagram is referenced to the free energy of the aggregates, which do not bind to peptide inhibitors under those conditions. The ground state of monomer is stabilized by peptides by 0.4 to 0.5 kcal/mol, greater than the stabilization of 0.2 kcal/mol for the ground state of tetramer caused by peptides. The transition state between tetramer and monomer is destabilized by 0.4 and 0.6 kcal/mol by TabF2/TabH2 and ffTAD1, respectively. The free energy of the transition state between monomer and aggregate is comparable in the presence and absence of the peptides.

### Peptide inhibitors bind to soluble oligomers at low temperature and acidic pH

We next probed how the peptide inhibitors affect the kinetics of formation of oligomers at 277 K and pH 4.4, an experimental condition known to slow down TTR aggregation and favor the formation of NMR-visible oligomeric ensembles (20). The NMR-visible oligomers show a broad peak with the ^19^F chemical shift between those for the tetramer and the monomer (18, 20). We have previously shown that under these conditions, there is ^19^F-NMR signal loss but no increase in turbidity at 330 nm over 3 days following a pH jump from pH seven to pH 4.4 (18). Similar results were obtained in the presence of the peptide inhibitors (Fig. S6), confirming no formation of insoluble TTR aggregates that scatter light at 330 nm after 3 days. Thus, both NMR-visible and NMR-invisible TTR oligomers are soluble at 277 K and pH 4.4 with and without peptides.

**Figure 5:**
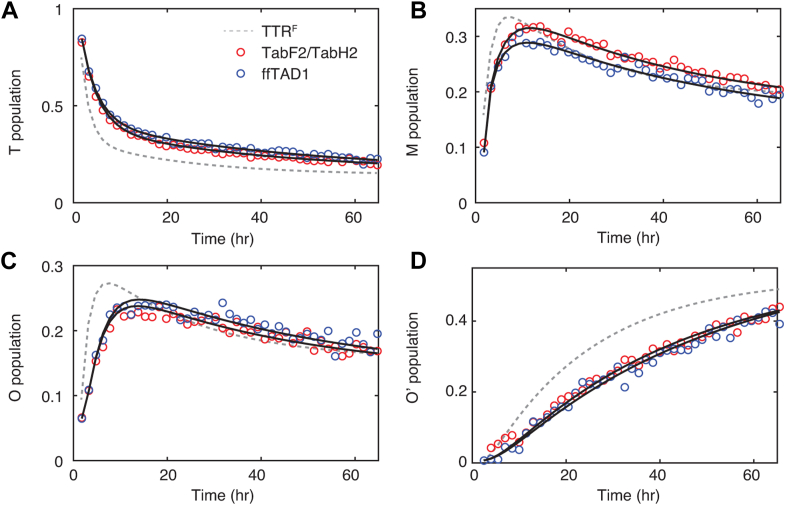
**^19^F aggregation kineticsat 277K.** Tetrameric TTR^F^ (2.5 μM) was incubated with 1 μM of each TabF2/TabH2 and 1 μM ffTAD1 at 277 K and pH 4.4. For clarity, open circles are measured data points for tetramer (*A*), monomer (*B*), NMR-visible oligomer (*C*), and NMR-invisible oligomer (*D*) signals with peptides. *Black lines* are fitted tetramer ↔ monomer↔ NMR-visible oligomer ↔ NMR-invisible oligomer models for data sets with peptides, and *dashed gray lines* are the four-state fits for the TTR^F^-only control to guide eyes.

Real-time ^19^F-NMR aggregation measurements were performed for 2.5 μM (tetramer concentration) TTR^F^ in the presence of 1 μM TabF2/TabH2 or 1 μM ffTAD1 at 277 K and pH 4.4. Compared to the TTR^F^-only control (dashed gray lines in Fig. 5*A*), TabF2/TabH2, and ffTAD1 slow the decay of the tetramer population and delay by 5 to 6 h the times at which the populations of monomer and NMR-visible oligomers reach their maximum (Fig. 5, *B* and *C*). The presence of the peptides also decreases the rate of formation of soluble, NMR-invisible oligomers (Fig. 5*D*). Fitting the aggregation data to a four-state model tetramer ↔ monomer ↔ NMR-visible oligomer ↔ NMR-invisible oligomer (20) allows extraction of six apparent rates (Table 2). In contrast with the aggregation rates at 310 K and pH 4.4 (Table 1), all six rates, including the disaggregation rate *k*_−3_, are reduced in the presence of peptides. TabF2/TabH2 and ffTAD1 are equally effective in inhibiting aggregation.

**Table 2:** Aggregation kinetics for TTR^F^ with and without peptides at pH 4.4 and 277 K

Condition	2.5 μM TTR^F^^a^	+ 1 μM TabF2/TabH2	+ 1 μM ffTAD1
*k*_1_ (h^−1^)^b^	0.37 ± 0.02	0.18 ± 0.01	0.17 ± 0.01
*k*_-1_ (h^−1^)^b^	0.28 ± 0.01	0.17 ± 0.01	0.19 ± 0.01
*k*_2_ (h^−1^)^b^	3.7 ± 0.4	2.4 ± 0.2	2.9 ± 0.2
*k*_-2_ (h^−1^)^b^	4.4 ± 0.5	3.1 ± 0.2	3.3 ± 0.2
*k*_3_ (h^−1^)^b^	0.074 ± 0.002	0.049 ± 0.001	0.043 ± 0.001
*k*_-3_ (h^−1^)^b^	0.022 ± 0.001	0.011 ± 0.001	0.009 ± 0.001

a Fitted parameters for TTR^F^ are taken from Ref (20) for comparison.

b The four-state kinetic model is, $T\begin{array}{c} k_{1}\\ ⇌\\ k_{-1} \end{array}M\begin{array}{c} k_{2}\\ ⇌\\ k_{-2} \end{array}O\begin{array}{c} k_{3}\\ ⇌\\ k_{-3} \end{array}O^{\prime }$, where T, M, O, and O′ stand for tetramer, monomeric intermediate, NMR-visible oligomers, and NMR-invisible oligomers, respectively, and six fitted parameters are first-order apparent aggregation rates for population conversions. The uncertainty in rates was calculated as one standard deviation from 50 bootstrap datasets.

To understand how peptide binding to distinct TTR species affects rates in the four-state kinetic model, we performed a series of four-state numerical simulations, where a reversible binding of an aggregation inhibitor was added to each of the four species. Analogous to the three-state binding kinetics simulations, the binding of peptides only reduces the rates of the outgoing processes that consume the respective species (Fig. S7). For example, binding of the peptide inhibitor to the NMR-invisible oligomeric species only reduces *k*_−3_. Collectively, our kinetic measurements show that at 277 K and pH 4.4, the peptides not only bind to the misfolded tetrameric and monomeric states but also bind to soluble NMR-visible and NMR-invisible TTR oligomers.

## Discussion

Understanding the mechanism by which designed peptides reduce the rate of TTR aggregation is critical for the development and optimization of novel aggregation inhibitors. Quantitative measurements of the population of aggregation intermediates in the multi-step aggregation pathway of TTR, enabled by the highly sensitive ^19^F-NMR aggregation assay, provide key mechanistic insights into the binding modes of the designed peptide inhibitors.

The designed peptides TabF2, TabH2, and their catenated version ffTAD1 selectively bind to monomeric TTR intermediates at acidic pH, stabilizing these species and modulating their population dynamics. The TabF2/TabH2 peptides were designed to bind the F and H β-strands of TTR (5, 6), which are solvent-accessible in the monomer but buried within the well-folded tetramer. At 310 K and pH 4.4, 1 μM each of TabF2 and TabH2 binds to the weakly populated monomeric aggregation intermediate, increasing the steady-state population from ∼300 nM to ∼400 nM (Table S1). ffTAD1, in which TabF2 and TabH2 are catenated into a single peptide chain, further increases the monomeric population to ∼500 nM at 1 μM. The weak binding (*K*_d_ > 100 μM) between ffTAD1 and a structured F87A monomer at pH 7.0 and 310 K (Fig. 2) is insufficient to inhibit TTR aggregation, as shown by numerical simulations (Fig. S8*A*).

Peptide binding to TTR is significantly enhanced at acidic pH, likely due to conformational destabilization of the monomer rather than electrostatic attraction. Lowering the pH below the isoelectric point increases the net positive charge of both the TTR protein and the peptides. For WT TTR, the net charge increases from −4.8 at pH 7.0 to +7.1 per protomer at pH 4.4. For the peptides, the net charge increases from +5.0 to +9.4 for TabF2/TabH2 (combined sequences) and from +5.4 to +14.3 for ffTAD1. Given the electrostatic repulsion, it is unlikely that electrostatics alone drive the enhanced binding between TTR monomers and peptides at acidic pH. Rather, localized misfolding of the monomeric intermediate at low pH, particularly at the F and H β-strands (19, 21), is more likely to contribute to the enhanced peptide-monomer interaction. At this pH, the monomeric intermediate retains a structured core with extensive β-sheets (20, 22). However, at pH 4.4, enhanced conformational fluctuations of the H strand expose highly amyloidogenic regions of β-strand G (20), potentially providing a binding site for the peptide inhibitors. Fully denatured TTR does not bind to the designed peptides (7).

Although the peptides also bind to TTR tetramers, their interactions are weak at neutral pH but more stabilized at acidic pH, suggesting that conformational changes in the tetramer promote binding. For the binding between peptides and the A TabF2/TabH2 mixture binds weakly (*K*_d_ > 100 μM) to the WT TTR tetramer at neutral pH resulting in small chemical shift perturbations (Fig. S2*A*). Chemical shift changes were observed for residues M13, A120, and V122, which are located near the central thyroxine-binding cavity. This suggests that parts of the peptides could enter the central hydrophobic cavity within the TTR tetramer, as previously observed for β-amyloid (23). At acidic pH, we observed a reduced dissociation rate (*k*_1_) with peptides compared to the TTR^F^ control (Table 1), indicating binding between the peptides and the tetramer. Numerical simulations further show that inhibitors with *K*_d_ = 100 μM for binding to both tetramer and monomer cannot inhibit TTR aggregation at the experimental concentrations (Fig. S8*B*). These results therefore suggest an enhanced binding between the peptides and the TTR tetramer at acidic pH and conformational misfolding at the tetramer level as well. Energetically, the peptide binding stabilizes the tetramer, though to a lesser extent than the monomer (Fig. 4). The stabilized complex of tetramer:peptide shows a reduced dissociation rate (*k*_1_). Likewise, the free-energy stabilization of the bound monomer:peptide complex slows down the forward aggregation rate (*k*_2_) to form insoluble aggregates, also contributing to the reduction in the reverse tetramerization rate (*k*_−1_) (Table 1).

Despite their inhibitory activity, the peptides do not bind preformed aggregates under standard conditions, suggesting that their effects are limited to early intermediates or to conformations that may only arise *in vivo*. At 310 K and pH 4.4, the disaggregation rate (*k*_−2_) in the three-state model tetramer ↔ monomer ↔ aggregate remains unchanged upon peptide addition (Table 1). Under these conditions, the loss of the ^19^F-NMR signal occurs at the same rate as the increase in turbidity (18), since the aggregates form quickly and precipitate. In these samples, oligomer formation is not observed, and the resulting aggregates are predominantly amorphous and non-fibrillar. These aggregates have previously been shown to exhibit low thioflavin T fluorescence compared to *in vitro* seeded fibrillar aggregates (5). The lack of change in *k*_-2_ indicates that the designed peptides are not able to bind amorphous aggregates formed *in vitro* at acidic pH. By contrast, TabF2/TabH2 (6) and ffTAD1 (14) bind to *ex vivo* TTR amyloids extracted from patients with transthyretin amyloidosis. These data suggest that aggregates formed at pH 4.4 and 310 K *in vitro* are conformationally distinct from *ex vivo* TTR amyloids, contradicting a prior report on structural comparison of the two forms of aggregates using low-resolution transmission electron microscopy and one-dimensional Fourier transform infrared spectroscopy (24).

Under slower aggregation conditions, the peptides can bind soluble oligomers, which may share structural features with *in vivo* TTR amyloids. At 277 K and pH 4.4, the disaggregation rate (*k*_−3_) in the four-state model tetramer ↔ monomer ↔ NMR-visible oligomer ↔ NMR-invisible oligomer is reduced in the presence of peptide inhibitors. At this lower temperature, aggregation of TTR is slowed, and oligomers can be detected with a time resolution of approximately 1 h (20). The significant decrease in *k*_−3_ shows that the designed peptides can bind soluble oligomers formed *in vitro* at acidic pH. Given that TAD1, the fluorophore-labeled version of ffTAD1, detects circulating transthyretin amyloid aggregates in plasma (14), our results suggest structural similarity between oligomers formed at 277 K/pH 4.4 *in vitro* and TTR amyloids formed *in vivo.* In addition, these results illustrate how TTR aggregate morphologies are affected by different experimental conditions (25, 26). In this regard, the condition-dependent TTR aggregation is similar to that of other amyloidogenic peptides, including α-synuclein (27), β-amyloid (28), huntingtin (29), and amylin (30), as the multi-step aggregation pathways are generally sensitive to experimental conditions, both *in vitro* and *in vivo* (3).

The observed sub-stoichiometric inhibition reflects the low abundance of aggregation intermediates relative to peptide concentration, enhancing inhibitory efficiency. We observed potent sub-stoichiometric inhibition of early TTR aggregation steps with 1 μM peptides for 2.5 μM TTR tetramer at 310 K and 277 K. At 310 K, the maximal molar concentration of the monomeric aggregation intermediate is less than 1 μM (Fig. 3, *B* and *C*). At 277 K, the NMR-soluble oligomers with at least 30 protomers (20) also show a maximal molar concentration less than 1 μM (Fig. 5*C*). Due to the lower concentrations of the intermediate species under both conditions, the binding stoichiometry between peptides and these species is greater than 1, which could lead to the observed sub-stoichiometric inhibition with respect to the peptide-to-tetramer molar ratio (1: 2.5).

In conclusion, we have elucidated the mechanism of inhibition of TTR aggregation by designed peptides TabF2/TabH2 and ffTAD1 using a combination of ^19^F-NMR aggregation assays and kinetic modeling. Our results confirm that the peptides bind and stabilize the monomeric aggregation intermediate, thereby slowing the forward aggregation rate. We show that peptides also bind to the misfolded tetramers and soluble oligomers. Our platform to study the inhibition mechanism for reversible aggregation is generalizable and complements the existing analysis for irreversible aggregation (31). Our methods could be extended to study aggregation pathways of other aggregation-prone proteins and develop aggregation inhibitors against these pathogenic species.

## Experimental procedures

### Protein expression and peptide production

The C10S-S85C TTR construct used in ^19^F aggregation assays was expressed, purified, and labeled with bromo-1,1,1-trifluoroacetone per Ref (18). The ^15^N-labeled wild-type and monomeric F87E TTR used in heteronuclear single quantum coherence (HSQC) experiments were expressed and purified using protocols described in Ref (32). The ^15^N source was ^15^N (NH_4_)_2_SO_4_ (1g/L). Peptide development was performed by rational design starting from peptide inhibitors that target the F and H strand segments of transthyretin (5, 6). Peptides were synthesized by LifeTein LLC in lyophilized form. All peptides were dissolved in 1-mM aliquots in Milli-Q water. Labeled proteins and peptides were frozen at −80 °C before use.

### Turbidity assay

The turbidity at 330 nm was measured for 2.5 μM (tetramer concentration) TTR^F^ with and without 1 μM peptides at 310 K/pH 4.4 and 277 K/pH 4.4 as previously described (1).

### NMR spectroscopy

^19^F-NMR spectra were acquired using Bruker Avance 600 or Avance 700 MHz spectrometers as previously described (18). The *T*_1_ of the TTR species is around 0.3 s (20) and thus, a 1-s recycle delay was used in our NMR experiments. The aggregation assays were performed in buffers containing 50 mM sodium acetate and 100 mM potassium chloride at pH 4.4. A trace amount of trifluoroacetic acid was included in NMR samples as an internal standard for referencing peak positions. The ^15^N-HSQC spectra of WT TTR and the F87E variant in a buffer containing 10 mM potassium phosphate and 100 mM KCl at pH 7.0 were acquired using the Bruker Avance 700 MHz spectrometer. Weighted average chemical shift perturbations (CSPs) for backbone amides were calculated using $\sqrt{{\left(\Delta \delta^{1}H\right)}^{2}+{\left(\Delta \delta^{15}N/5\right)}^{2}}$, where Δδ means chemical shift differences for either ^1^H or ^15^N with and without peptides.

### Numerical modeling for reversible inhibitor binding

Reversible steps (binding and unbinding of inhibitors) are added to each of the applicable TTR species (tetramer, monomer, and aggregate in the reversible three-state fits or tetramer, monomer, NMR-visible oligomer, NMR-invisible oligomer in the four-state fits) in an expanded kinetic model. Previously published rates for TTR^F^ aggregation at 310 K and pH 4.4 (18) or 277 K and pH 4.4 (20) were used in the simulations. The apparent binding affinity between an inhibitor and the TTR species, whose relative populations are based on relative protomer concentrations or normalized peak areas from the ^19^F-NMR experiments, was set at 100 nM. An off-rate constant of 100 h^−1^ was used in simulations, which is physically reasonable given the apparent binding affinity used in simulations (33). The ordinary differential equations were solved by *ode23* or *ode15s* in custom MATLAB codes.

## Data availability

All data are contained within the manuscript and the supporting information.

## Supporting information

This article contains supporting information. Full HSQC spectra of WT and F87E titration with TabF2/TabH2, Normalized NMR signal for TTR^F^ with TabH2, TabF2, or their equal-molar combination, ^19^F spectra of TTR^F^ with and without TAD1, turbidity assays at pH 4.4/277 K and pH 4.4/310 K with and without 1 μM peptides, three-state or four-state aggregation kinetic simulations with inhibitors, and simulated aggregation kinetics of TTR^F^ in the presence of an inhibitor with *K*_d_ = 100 μM. Pseudo steady-state populations of TTR species at pH 4.4/310 K with and without peptide inhibitors.

## Conflict of interest

The authors declare the following financial interests/personal relationships which may be considered as potential competing interests: Dr Saelices reports advisory board, speaker, and consulting fees from Alexion, Pfizer, Attralus, Intellia, and AmyGo. Other authors declare that they have no conflicts of interest with the contents of this article.
