# Supplementary material for: Mechanism of inhibition of acid-mediated transthyretin aggregation by designed peptides
Source: J Biol Chem. 2025 Aug 13;301(10):110594. doi: 10.1016/j.jbc.2025.110594 (PMC12624794; doi:10.1016/j.jbc.2025.110594)
Supplement: Supporting information [file mmc1.docx]

Supplementary Information for

**Mechanism of inhibition of acid-mediated transthyretin aggregation by designed peptides**

Xun Sun^1^, Rose Pedretti^2^, H. Jane Dyson^1^, Lorena Saelices^2^, Peter E. Wright^1^

1. Department of Integrative Structural and Computational Biology and Skaggs Institute of Chemical Biology, The Scripps Research Institute, La Jolla, CA, U.S.

2. Center for Alzheimer’s and Neurodegenerative Diseases, Department of Biophysics, Peter O’Donnell Jr Brain Institute, University of Texas Southwestern Medical Center, Dallas, TX, U.S.

Corresponding author: [wright@scripps.edu](mailto:wright@scripps.edu)

## Table S1. Pseudo steady-state populations of TTR species with and without peptide inhibitors at pH 4.4 and 310 K.

| Population (%) | Tetramer ^a^ | Monomer ^a^ | Aggregates ^a^ |
| --- | --- | --- | --- |
| 2.5 µM TTR^F^ | 20.2 ± 1.5 | 2.7± 0.2 | 77.1 ± 1.5 |
| + 1 µM TabF2/TabH2 | 24.6 ± 1.7 | 4.4 ± 0.3 | 71.0 ± 1.6 |
| + 1 µM ffTAD1 | 25.3 ± 1.5 | 5.2 ± 0.3 | 69.5 ± 1.4 |

^a^ Populations were derived from fitted rates from the reversible tetramer$\leftrightarrow$monomer$\leftrightarrow$aggregate kinetic model (Table 1). Tab2s denotes a mixture of equal concentrations of TabF2 and TabH2. The tetramer, monomer, and aggregate populations are calculated by $k_{-1}k_{-2}/D$, $k_{1}k_{-2}/D$, and $k_{1}k_{2}/D$, where the common normalizing denominator is *D* $=k_{1}k_{2}+k_{1}k_{-2}+k_{-1}k_{-2}$ (See Table 1 legends for the rate notations). The error bar is plotted as 1 standard deviation from 50 bootstrapped datasets.


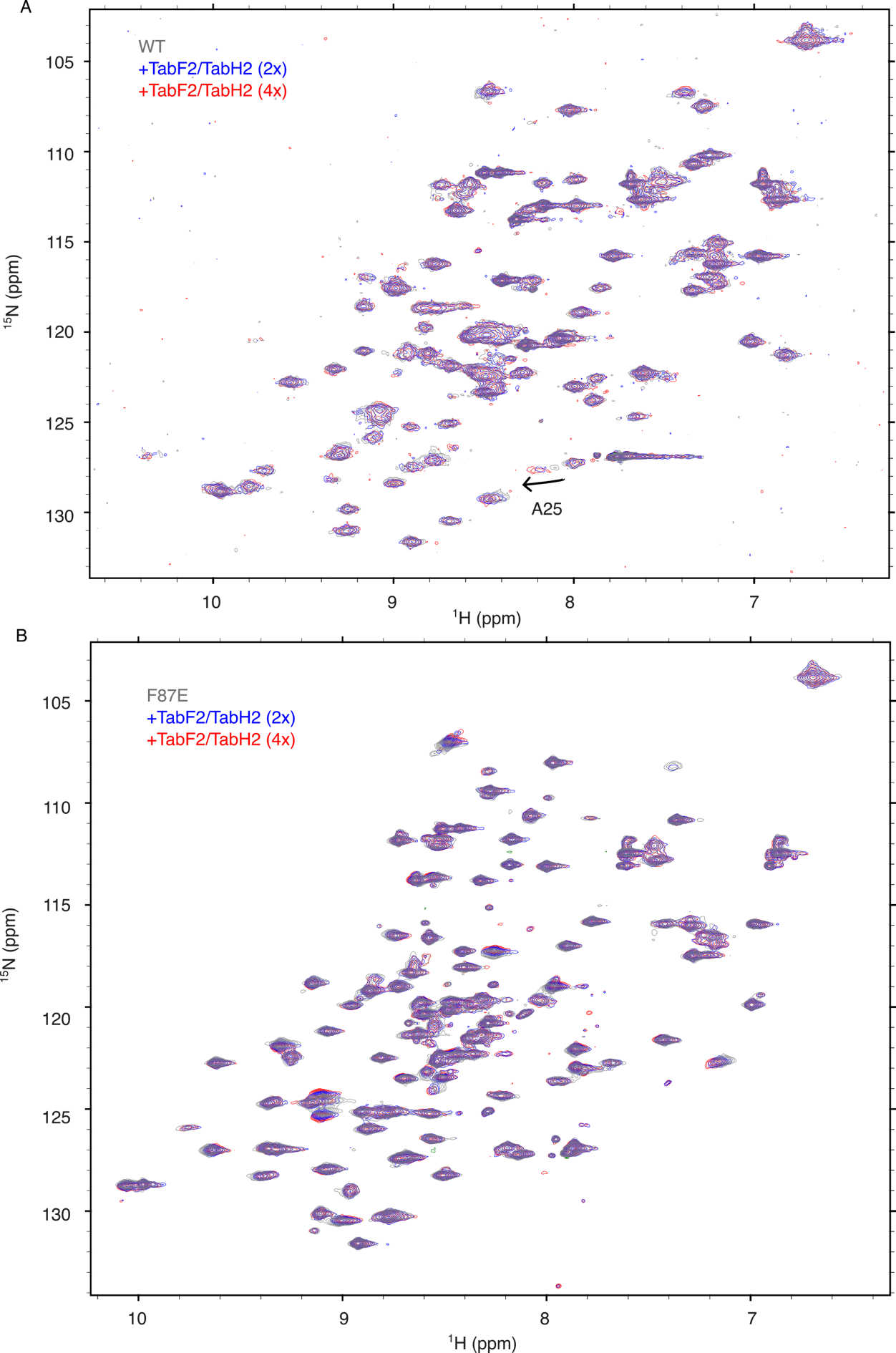


Figure S1. ^15^N-HSQC spectra of WT (A) and F87E (B) with equal concentrations of TabF2/TabH2 titrated at the (individual peptide: TTR) molar ratios labeled. Spectra were collected at 298 K and pH 7.0 in NMR buffer for 56 µM (tetramer concentration) for WT (A) and 106 µM (protomer concentration) for monomeric F87E (B).


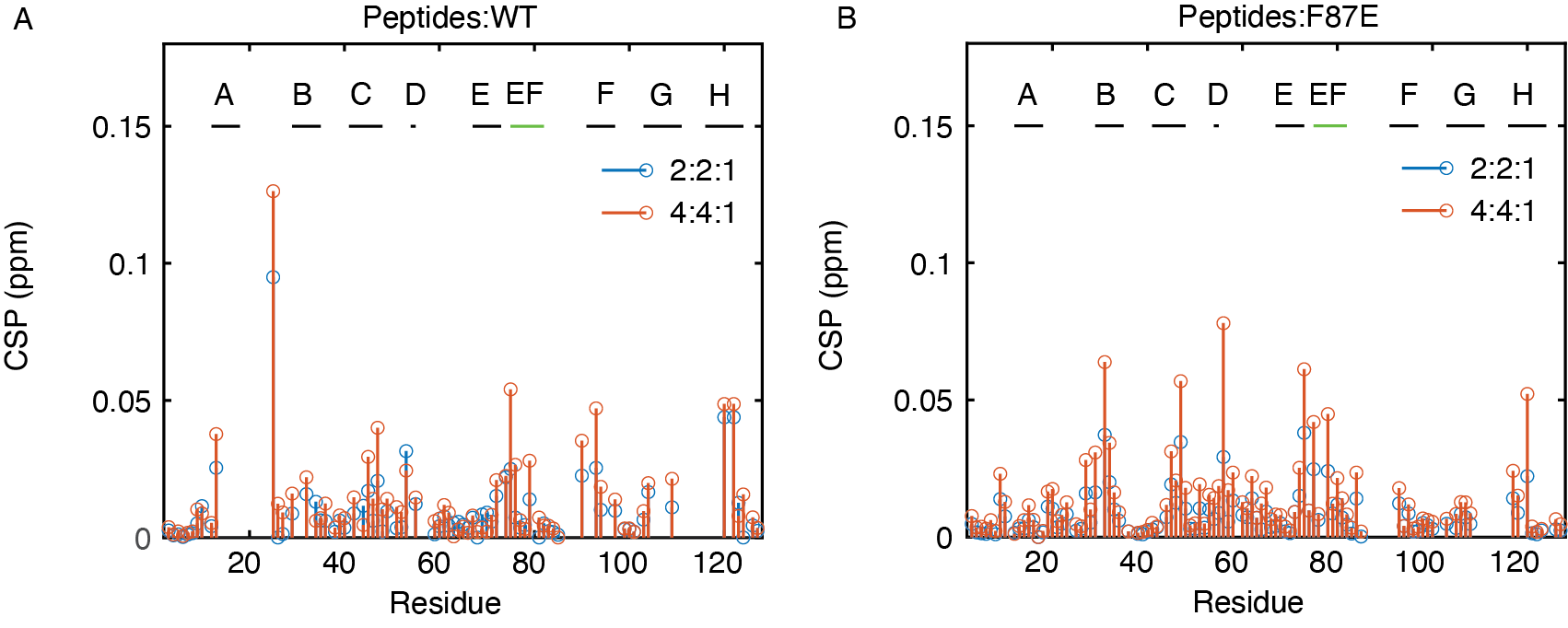


Figure S2. Weighted average chemical shift perturbations (CSPs) of backbone amides from HSQC spectra for WT (A) and F87E (B) with equal concentrations of TabF2 and TabH2 titrated at 2-fold and 4-fold molar ratios to TTR at 298 K and pH 7.0.


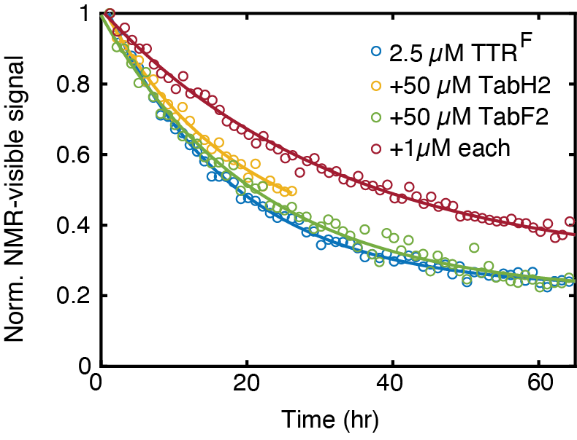


Figure S3. Normalized total NMR-visible signal for 2.5 µM TTR^F^ alone (tetramer concentration), with 50 µM TabH2, 50 µM TabF2, and a combination of 1 µM TabH2 and 1 µM TabF2 at 310 K and pH 4.4. Colored solid lines are single exponential fits.


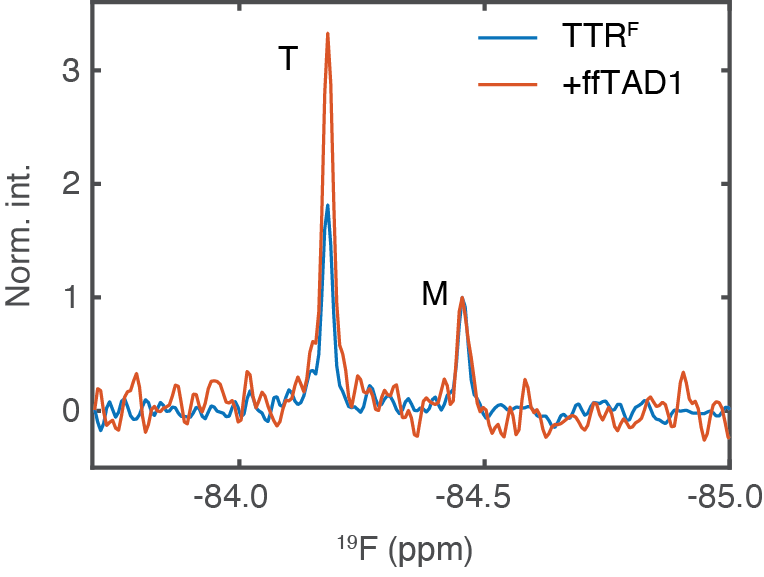


Figure S4. ^19^F-NMR spectra of 1.25 µM TTR^F^ (tetramer concentration) with and without 5 µM ffTAD1 at 298 K and pH 4.4. Spectra are normalized by the peak height of the monomer species. The minor changes in peak positions and peak widths of the tetramer and monomer species of TTR^F^ with and without ffTAD1 are less than 0.01 ppm and 1 Hz, respectively.


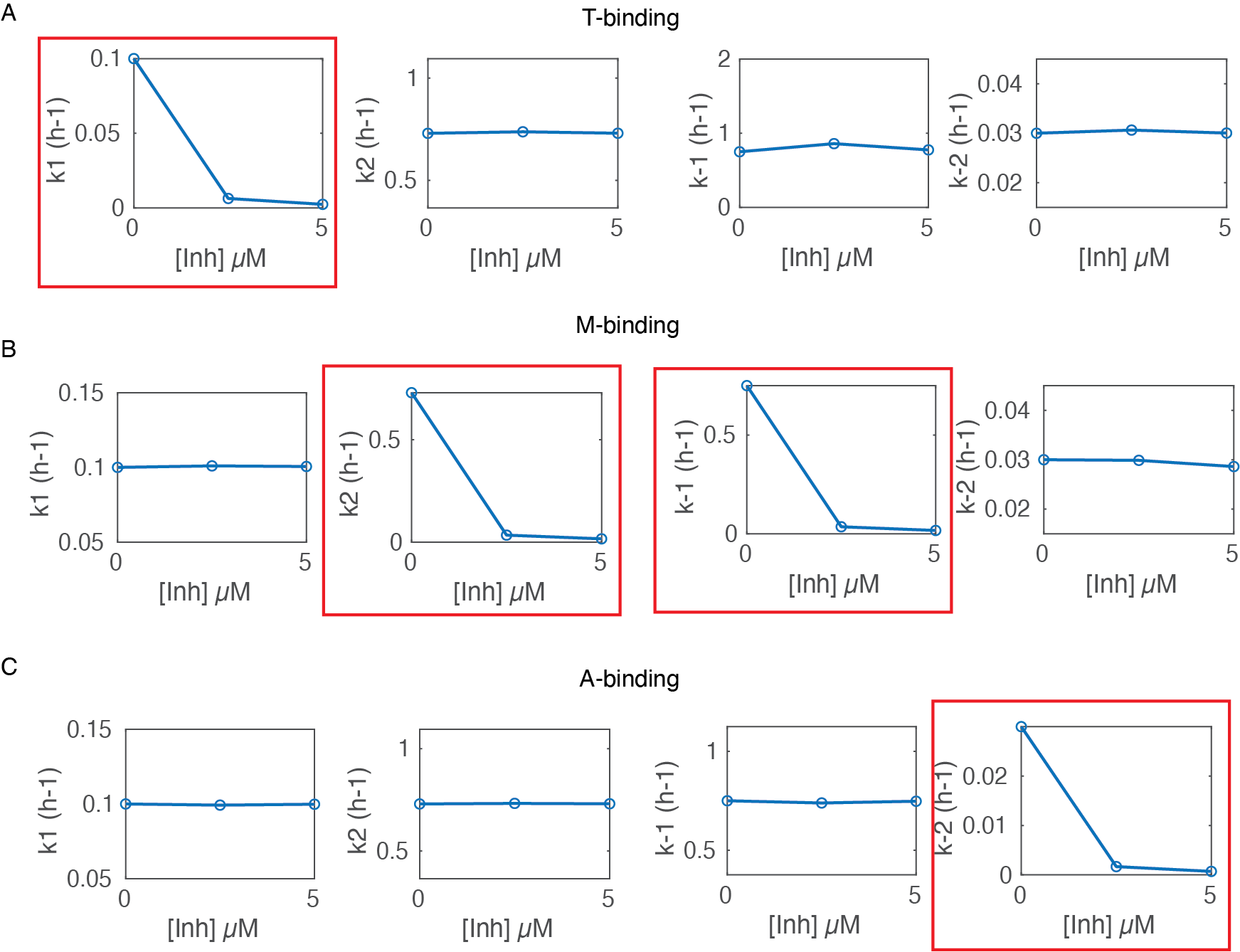


Figure S5. Numerical simulations of the three-state (T↔M↔A) kinetic model with inhibitors binding to tetramer (A), monomeric aggregation intermediate (B), and NMR-invisible aggregate (C) states at 310 K and pH 4.4. Each grouped panel with four subplots shows how the reversible inhibitor binding changes one of the four fitted rates (*k*_1_, *k*_-1_, *k*_2_, and *k*_-2_) as a function of the inhibitor concentration. The subplots of specific rates reduced by the inhibitor binding are highlighted using red boxes.


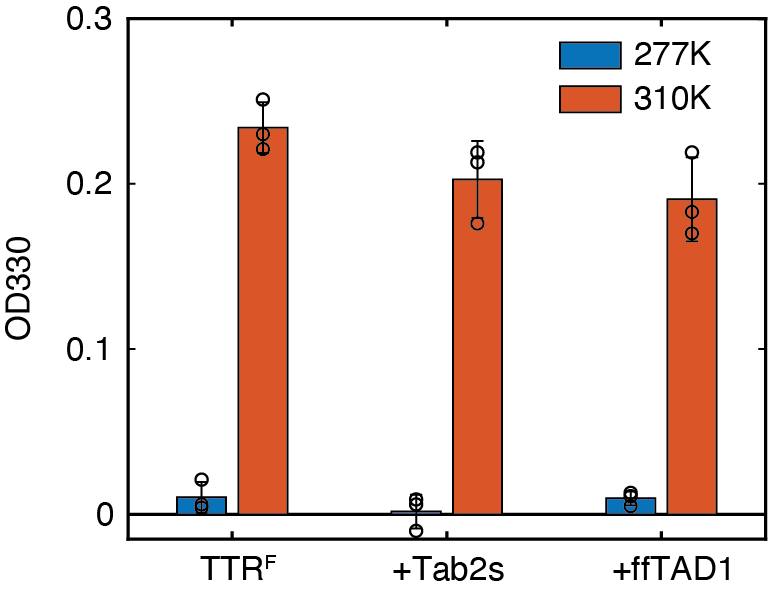


Figure S6. Turbidity at 330 nm (OD330) for 2.5 µM TTR^F^ (tetramer concentration) with and without 1 µM peptides at 310 K/pH 4.4 or 277 K/pH 4.4. Tab2s denotes a mixture of equal concentrations of TabF2 and TabH2. The samples were incubated without agitation for 3 days before the turbidity reading.


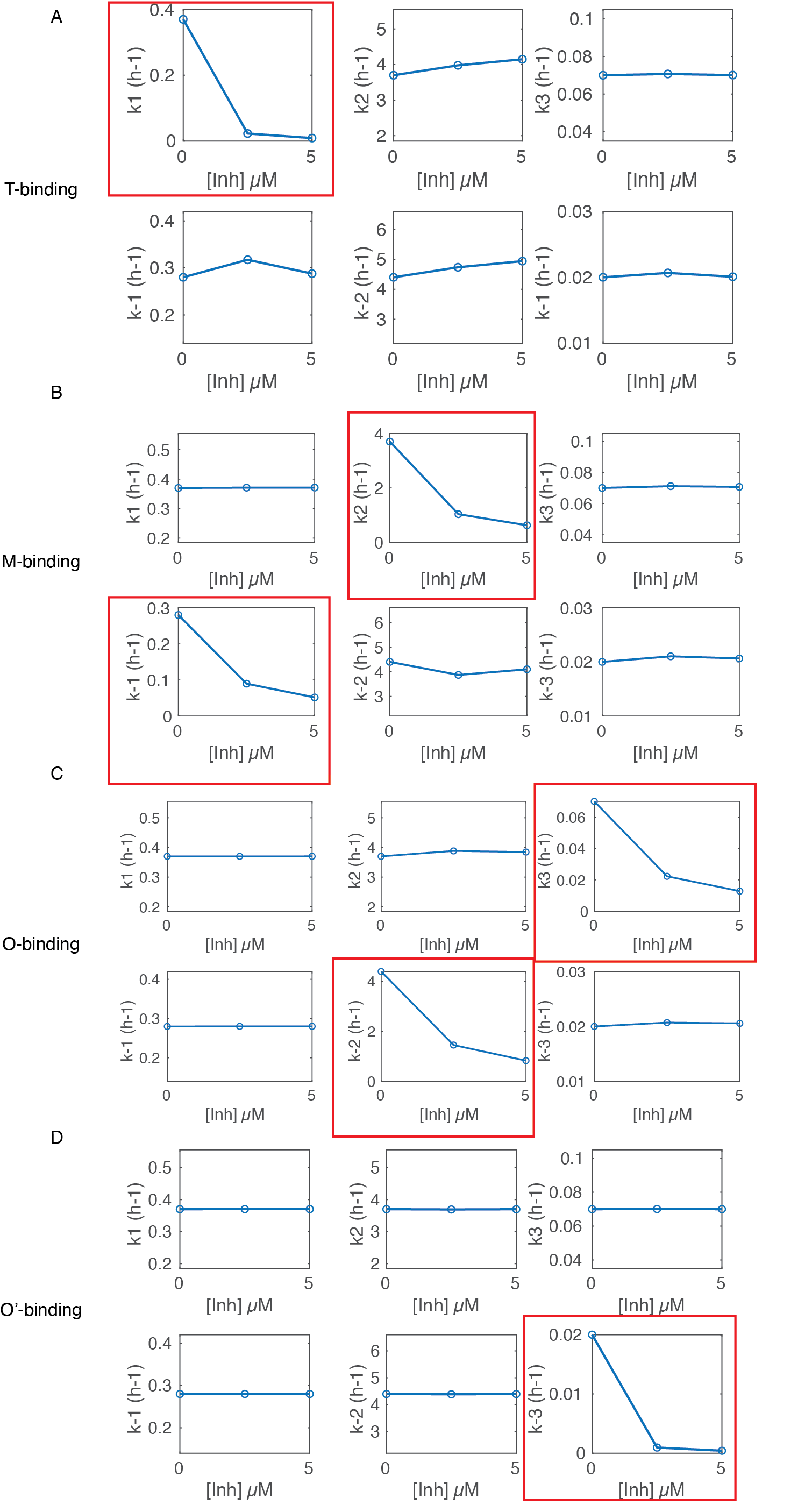


Figure S7. Numerical simulations of the four-state (tetramer ↔ monomer ↔ NMR-visible oligomer ↔ NMR-invisible oligomer) kinetic model with inhibitors binding to tetramer (A), monomeric aggregation intermediate (B), NMR-visible oligomer (C), and NMR-invisible oligomer (D) states at 277 K and pH 4.4. Each grouped panel with six subplots shows how the reversible inhibitor binding changes one of the six rates (*k*_1_, *k*_-1_, *k*_2_, *k*_-2_, *k*_3_, and *k*_-3_) as the concentration of the inhibitor increases. The subplots of specific rates reduced by the inhibitor binding are highlighted using red boxes.


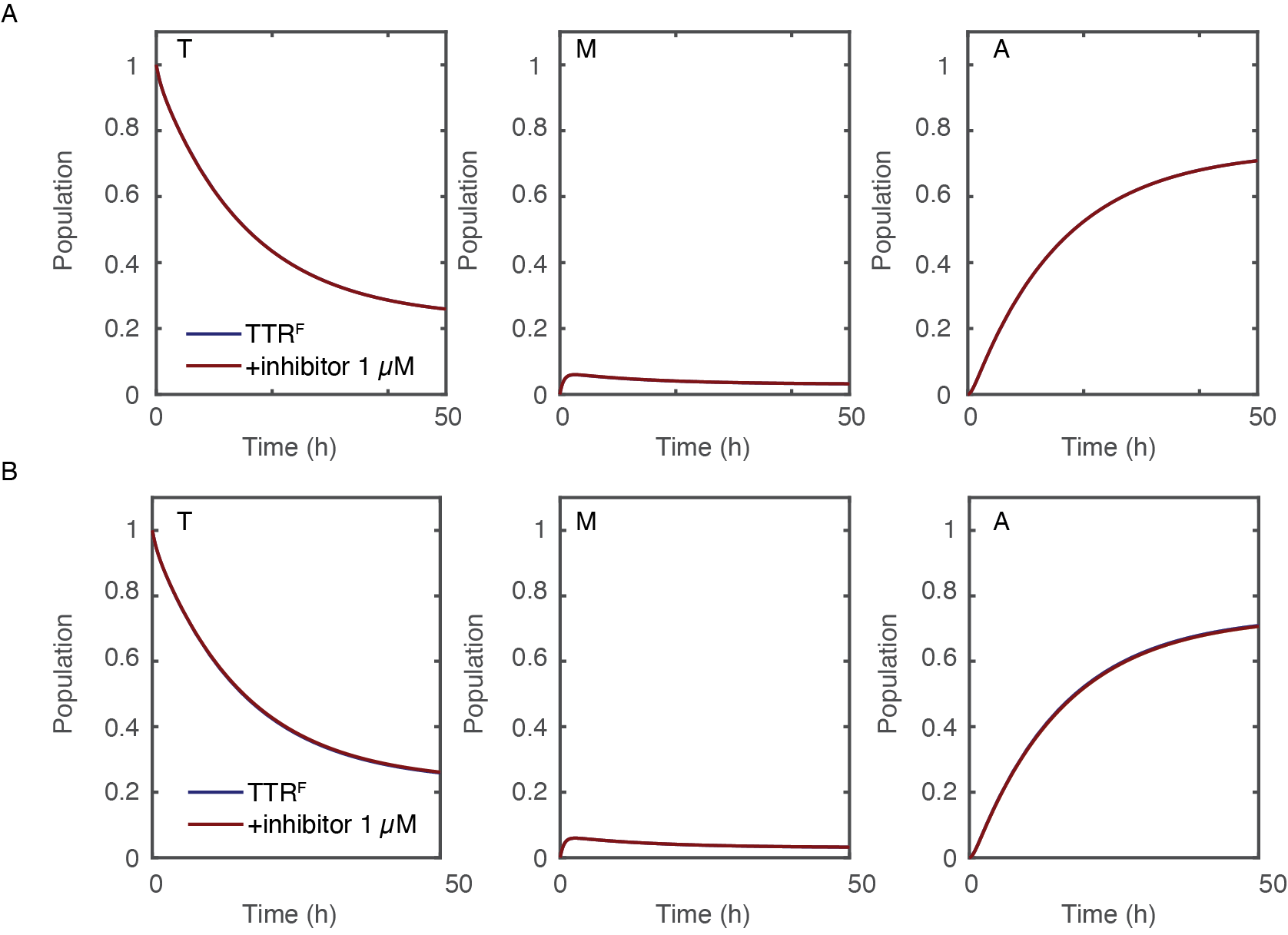


Figure S8. Numerical simulations show that an aggregation inhibitor at 1 µM with a *K*_d_ = 100 µM for only monomer (A) and both tetramer and monomer species (B) is ineffective against 2.5 µM (tetramer concentration) TTR^F^ aggregation at 310 K and pH 4.4. In both cases, the time-dependent population curves for TTR^F^ alone (blue) overlap with those representing TTR^F^ and 1 µM inhibitor (dark brown).
